# Is tea consumption associated with the serum uric acid level, hyperuricemia or the risk of gout? A systematic review and meta-analysis

**DOI:** 10.1186/s12891-017-1456-x

**Published:** 2017-02-28

**Authors:** Yi Zhang, Yang Cui, Xuan-an Li, Liang-jun Li, Xi Xie, Yu-zhao Huang, Yu-hao Deng, Chao Zeng, Guang-hua Lei

**Affiliations:** 1 Department of Orthopaedics, Xiangya Hospital, Central South University, No.87 Xiangya Road, Changsha, Hunan Province 410008 China; 2 Department of International Medical Service, Xiangya Hospital, Central South University, Changsha, Hunan Province 410008 China

**Keywords:** Tea, Serum uric acid, Hyperuricemia, Gout, Systematic review, Meta-analysis

## Abstract

**Background:**

The aim of this study was to examine the associations of tea consumption with the serum uric acid (SUA) level, hyperuricemia (HU) and the risk of gout.

**Methods:**

A comprehensive literature search up to June 2016, using PUBMED and EMBASE databases, was conducted to identify the relevant observational studies that examined the associations of tea consumption with the SUA level, HU and the risk of gout.

**Results:**

A total of fifteen observational studies were included in this study, and nine studies were extracted for meta-analysis. For the SUA level, seven studies were included. According to the combined weighted mean difference (WMD), there was no significant difference between the highest and the lowest tea intake category in terms of the SUA level (WMD = 7.41 μmol/L, 95%CI: −2.34 to 17.15; *P =* 0.136). In subgroup analysis including three studies, green tea consumption was positively associated with the SUA level (WMD = 17.20 μmol/L, 95%CI: 7.00 to 27.40; *P =* 0.01). For the prevalence of HU, five studies were included. The overall multi-variable adjusted odds ratio (OR) for the highest versus the lowest category of tea consumption was 0.98 (95%CI: 0.77 to 1.24; *P =* 0.839). For the risk of gout, two prospective cohort studies showed that there was no relationship between tea consumption and the risk of gout in males and females, respectively.

**Conclusion:**

The current evidences suggest that tea consumption does not seem to be associated with the SUA level, HU and the risk of gout. However, due to the limited number of studies, green tea consumption might be positively associated with the SUA level. More well-designed prospective cohort studies are needed to elaborate these issues further.

**Electronic supplementary material:**

The online version of this article (doi:10.1186/s12891-017-1456-x) contains supplementary material, which is available to authorized users.

## Background

Hyperuricemia (HU) is a major cause of disability, which has drawn increasing attention in recent decades because of its high prevalence in the global context [1–3]. HU occurs when the concentration of serum uric acid (SUA), determined by the production and excretion of urate, exceeds a normal standard. Epidemiological findings have shown that around 21.4% of American adults suffer from HU [4], while the prevalence of HU in some Asian countries ranges from 13 to 25.8% [5–9]. Emerging data indicated that HU can increase the risk of hypertension, cardiovascular disease, diabetes and chronic kidney disease [10–13]. HU is also known as the precursor of gout, the most common inflammatory arthritis in adult men [14]. In the presence of SUA concentration above saturation levels (≥410 mmol/L, 6.8 mg/dL), monosodium urate (MSU) crystals form at physiological temperature and pH [15]. The host response to MSU crystals leads to the clinical manifestations of gout, such as acute flares and tophaceous disease [16, 17]. There are several risk factors for gout, including obesity [18], hypertension [19] and certain aspects of diet, including the intake of alcohol [20] and purine-rich foods [21]. However, the specific pathogenesis of HU and gout has not yet been fully elucidated. Both the American College of Rheumatology (ACR) and the European League Against Rheumatism (EULAR) guidelines for the management of gout support diet modification alongside pharmacologic interventions [22, 23]. Thus, identifying the modifiable dietary factors for HU or gout appears to be an important step in the prevention and management of these conditions.

Tea, derived from the leaves of the Camellia sinensis plant, is one of the most popular beverages consumed worldwide [24], especially in Eastern European and Asian countries [25]. Tea contains several kinds of antioxidants including flavonoid, catechin, thearubigin and theaflavin [26]. It is noteworthy that tea is negatively associated with depression, cancer, Parkinson’s disease and cardiovascular disease [24, 25, 27, 28]. Several studies have reported that green tea extracts may decrease SUA levels in animals [29–31]. Therefore, a similar effect in humans may influence the prevalence of HU or gout, but current research from epidemiological studies remains unclear [32–38]. Thus, the present systematic review and meta-analysis of observational studies aimed at investigating the associations of tea consumption with the SUA level, HU and the risk of gout. It was hypothesized that tea consumption is inversely associated with the SUA level, HU and the risk of gout.

## Methods

### Search strategy

This systematic review and meta-analysis was performed with referencing to the Preferred Reporting Items for Systematic review and Meta-analyses (PRISMA) statement [39]. The electronic databases of PUBMED and EMBASE were searched up to June 2016, using a series of logic combinations of keywords and in-text words that are related to uric acid (‘uric acid’, ‘gout’, ‘hyperuricemia’, ‘urate’, ‘hyperuricaemia’) and tea (‘tea’). The search string is included in supplementary material (Additional file 1). No language restriction was imposed. The references of the retrieved articles and reviews were evaluated.

### Study selection

Two researchers (YZ and GHL) reviewed the titles, abstracts and full texts of all retrieved studies independently. Disagreements, if any, were resolved by discussions and mutual-consultations. All eligible studies should meet the following criteria: 1) observational studies (case–control, cohort or cross-sectional study); 2) the exposure of interest was tea; 3) the outcome of interest was the SUA level, the prevalence of HU and the risk of gout. The exclusion criteria were as follows: 1) duplicated or irrelevant articles; 2) reviews, letters, case reports or non-human studies; 3) inaccessibility of full-text.

### Data extraction

The available information and outcomes of each study were screened by the two researchers (YZ and GHL) independently. The data to be extracted were the first author, year of publication, location, age, gender, sample size, study design, exposure definition, original SUA value, OR for HU or RR for gout, type of tea, adjustments and outcomes. The primary outcome of interest was the difference in SUA concentration between the highest and the lowest category of tea consumption. The secondary outcome of interest was the odds ratio (OR) for the prevalence of HU and the relative risk (RR) for the risk of gout, for the highest versus the lowest category of tea consumption.

### Quality assessment

The methodological quality of included studies was evaluated in accordance with the Newcastle-Ottawa Scale (NOS) [40], which is developed for assessing the quality of non-randomised studies based on three broad perspectives: the selection of study groups; the comparability among different groups; and the ascertainment of either the exposure or outcome of interest. Disagreements with respect to the methodological quality of results, if any, were resolved by discussion and mutual-consultation.

### Statistical analyses

The outcome measures investigated in this meta-analysis were the SUA level and OR for the prevalence of HU. The weighted mean difference (WMD) and its corresponding 95% confidence interval (CI) for SUA were calculated respectively. The pooled OR of HU and its related 95%CI were also calculated. However, the pooled RR of gout and its related 95%CI were not calculated due to the limited number of studies (only two). Hence, their findings [33, 35] were simply reported in this result, respectively. The most multivariable adjusted OR values reported in the original study were extracted for calculation if more than one was reported. The homogeneity of effect size across trials was tested by Q statistics (*p <* 0.05 was considered heterogeneous). The random effect models were used for all the analysis. The I^2^ statistic, which measures the percentage of the total variation across studies due to heterogeneity, was also examined (I^2^ < 25% was considered low heterogeneity, I^2^ around 50% was considered moderate heterogeneity, I^2^ > 75% was considered high heterogeneity). Begg’s tests were performed to assess the publication bias [41], and statistical analyses were performed using STATA version 11.0 (StataCorp LP, College Station, Texas). A p value equal to or less than 0.05 was considered to be statistically significant, unless otherwise specified.

## Results

### Literature search and study characteristics

**Fig. 1:**
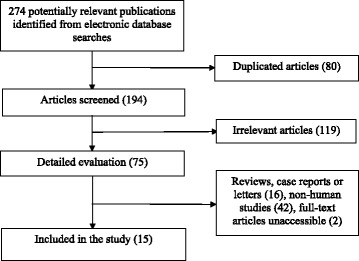
Flow chart for the identification of the included studies

The flow chart for the identification of the included studies was presented in Fig. 1. A total of two hundred and seventy four potentially relevant publications were retrieved during the initial literature search. After eliminating eighty duplicated articles, one hundred and ninety four articles were identified for detailed evaluation. One hundred and nineteen studies were excluded initially. Then, sixteen reviews, case reports or letters, forty two non-human studies, and two articles without full-text accessibility were removed [42, 43]. All of the excluded articles are listed in Additional file 2. Eventually, ten cross-sectional, one case–control and four cohort studies were included in this systematic review and meta-analysis. Eleven, five and two studies were related to the associations of tea consumption with the SUA level, HU and the risk of gout, respectively. Table 1 summarizes the main characteristics of these fifteen included studies. The methodological qualities of these studies were shown in Additional file 3: Table S1 (cross-sectional study), Table S2 (cohort study) and Table S3 (case–control study).

**Table 1:** Characteristics of the individual studies included in this systematic review and meta-analysis

First author year of publication	Location	Age years	Male (%)	Sample Size	Study design	Exposure definition	Original SUA value (umol/L), OR for HU or RR for gout (95%CI)	Type of tea	Adjustments	Outcome
David Curb 1986 [48]	USA	54.1	100	5858	Cohort	0 cups/day 1–2 cups/day 3–4 cups/day > 5 cups/day	Not mentioned	Not mentioned	NA	SUA
Tang 1998 [44]	China	58.3	75	416	Cross-Sectional	0 gram/day ≤5 grams/day >5 grams/day	325.4 (312.1–338.7) 339.4 (326.1–352.7) 354.2 (339.1–369.3)	Not mentioned	NA	SUA
Kiyohara 1999 [32]	Japan	52.0	100	2240	Cross-Sectional	<1 cups/day 1–2 cups/day 3–4 cups/day >5 cups/day	335.7 (327.4–344.6) 344.6 (336.9–353.0) 341.1 (333.9–348.8) 339.9 (332.1–347.6)	Green tea	Hospital, age, serum total cholesterol, serum HDL-cholesterol, serum creatinine, systolic blood pressure, BMI, rank, beer, alcohol, smoking status, meat, dairy products.	SUA
Yuan 2000 [45]	Taiwan	NA	NA	96	Case–control	Never, ever	288.1 (255.3–320.9) 200.0 (166.8–233.2)	Not mentioned	NA	SUA
Haldar 2007 [50]	Northern Ireland	18–64	31.5	89	Cross-Sectional	Never, ever	Not mentioned	Green tea and black tea combined	Age, sex and BMI	SUA
Choi 2007 [33]	USA	54.0	100	45869	Cohort	0 cups/day <1 cups/day 1–3 cups/day ≥4 cups/day	1.0 1.09 (0.92–1.30) 1.06 (0.85–1.33) 0.82 (0.38–1.75)	Not mentioned	Age, total energy intake, BMI, diuretic use, history of hypertension, history of renal failure, intake of alcohol, total meats, seafood, purine-rich vegetables, dairy foods, total vitamin C, decaffeinated coffee.	Gout (gout criteria of the American College of Rheumatology)
Choi 2007 [34]	USA	45.0	46.8	14314	Cross-Sectional	SUA: 0 cups/day <1 cups/day 1–3 cups/day ≥4 cups/day HU: 0 cups/day <1 cups/day 1–3 cups/day ≥4 cups/day	319.1 (317.6–320.6) 317.3 (315.5–319.0) 321.7 (317.3–326.2) 318.6 (312.8–324.4) 1.0 Not mentioned Not mentioned 1.00 (0.65–1.53)	Not mentioned	Age, sex, smoking status, BMI, smoking; use of diuretics, beta-blockers, allopurinol, uricosuric agents, hypertension, glomerular filtration rate, alcohol, total meats, seafood, dairy foods, decaffeinated coffee.	SUA HU (serum uric acid level >6.0 mg/dl)
Choi 2010 [35]	USA	46.0	0	89433	Cohort	0 cups/day <1 cups/day 1–3 cups/day ≥4 cups/day	1.0 1.05 (0.86–1.28) 0.92 (0.74–1.16) 1.55 (0.98–2.47)	Not mentioned	Age, total energy intake, BMI, menopause, use of hormonal replacement, diuretic use, history of hypertension, intakes of alcohol, sugar-sweetened soft drinks, total meats, seafood, chocolate, dairy foods, total vitamin C, decaffeinated coffee.	Gout (gout criteria of the American College of Rheumatology)
Yu 2010 [51]	China	40.2	48.4	7403	Cross-Sectional	Never, ever	1.0 0.84 (0.82–0.86)	Not mentioned	NA	HU (serum uric acid >7.0 mg/dl in males and >6.0 mg/dl in females)
Chang 2012 [47]	Taiwan	75	100	361	Cross-Sectional	Never, ever	Not mentioned	Not mentioned	NA	SUA
Teng 2013 [36]	Singapore	57.6	44.3	483	Cross-Sectional	SUA (Green tea): Non-drinkers, Monthly drinkers, Weekly drinkers, Daily drinkers. SUA (Black tea): Non-drinkers, Monthly drinkers, Weekly drinkers, Daily drinkers. HU (Green tea): Non-drinkers, Monthly drinkers, Weekly drinkers, Daily drinkers. HU (Black tea): Non-drinkers, Monthly drinkers, Weekly drinkers, Daily drinkers.	309.5 (298.4-321.0) 309.5 (290.4–329.9) 311.6 (296.3–327.7) 334.5 (316.2–353.9) 312.4 (301.4–323.8) 308.8 (289.0–329.9) 314.3 (298.6–330.7) 318.4 (299.6–338.4) 1.0 0.84 (0.36–1.98) 1.15 (0.62–2.14) 2.12 (1.03–4.36) 1.0 0.68 (0.28–1.67) 1.27 (0.68–2.37) 0.56 (0.25–1.27)	Green tea, black tea	Cholesterol, creatinine, HbA1c, triglycerides, age, gender, BMI, education, cigarette smoking status, physical activity status, hypertension at baseline, dairy products, red meat, fish, alcohol, soda, fruit juice.	SUA HU (serum uric acid level >6 mg/dl)
Chatzistamatiou 2015 [49]	Greece	51	53	660	Cross-Sectional	Never, ever	Not mentioned	Not mentioned	NA	HU (serum uric acid >7.2 mg/dl in males and >6.1 mg/dl in females)
Bae 2015 [37]	Korea	61.9	37.9	9400	Cross-Sectional	SUA (Male): <0.1 ml/day, 0.1–8.0 ml/day, 8.1–51.4 ml/day, ≥51.5 ml/day; SUA (Female): <0.1 ml/day, 0.1–8.0 ml/day, 8.1–51.4 ml/day, ≥51.5 ml/day; HU (Male): <0.1 ml/day, 0.1–4.0 ml/day, 4.1–25.7 ml/day, ≥51.5 ml/day; HU (Female): <0.1 ml/day, 0.1–4.0 ml/day, 4.1–25.7 ml/day, ≥51.5 ml/day;	345.0 (340.9-349.1) 336.9 (328.6–345.8) 338.7 (332.1–344.0) 348.1 (341.5–354.7) 260.6 (258.2–263.0) 262.0 (254.8–269.0) 261.3 (257.7–264.9) 263.6 (259.4–267.8) 1.0 0.92 (0.66–1.28) 0.95 (0.73–1.22) 1.27 (0.96–1.67) 1.0 0.97 (0.66–1.42) 0.84 (0.62–1.14) 1.13 (0.81–1.59)	Not mentioned	Age, education, marital status, cigarette smoking, alcohol drinking, regular exercise, BMI, triglyceride, fasting serum glucose, hypertension medication, glomerular filtration rate, total energy, vitamin c, meat intake, seafood intake, dairy food intake, soft drink intake, added sugar in coffee, added cream in coffee.	SUA HU (serum uric acid >7.0 mg/dl in males and >6.0 mg/dl in females)
Li 2015 [38]	China	37.7	52.5	1372	Cross-sectional	<1 time/week 1–6 times/week ≥6 times/week	1.0 0.74 (0.47–1.17) 0.56 (0.33–0.93)	Not mentioned	Age, smoking and drinking status	HU (SUA >416 μmol/L in males and >357 μmol/L in females)
Tian 2016 [46]	China	63	44.1	19471	Cohort study	Never, ever	280.0 (278.6–281.3) 301.1 (299.2–303.0)	Green tea	Age, sex, BMI, education, smoking status, alcohol, drinking status, physical activity, hypertension status, hyperlipidemia status, diabetes status and family history of CHD	SUA

### Weighted mean difference of SUA concentration between the highest and the lowest tea intake category

**Fig. 2:**
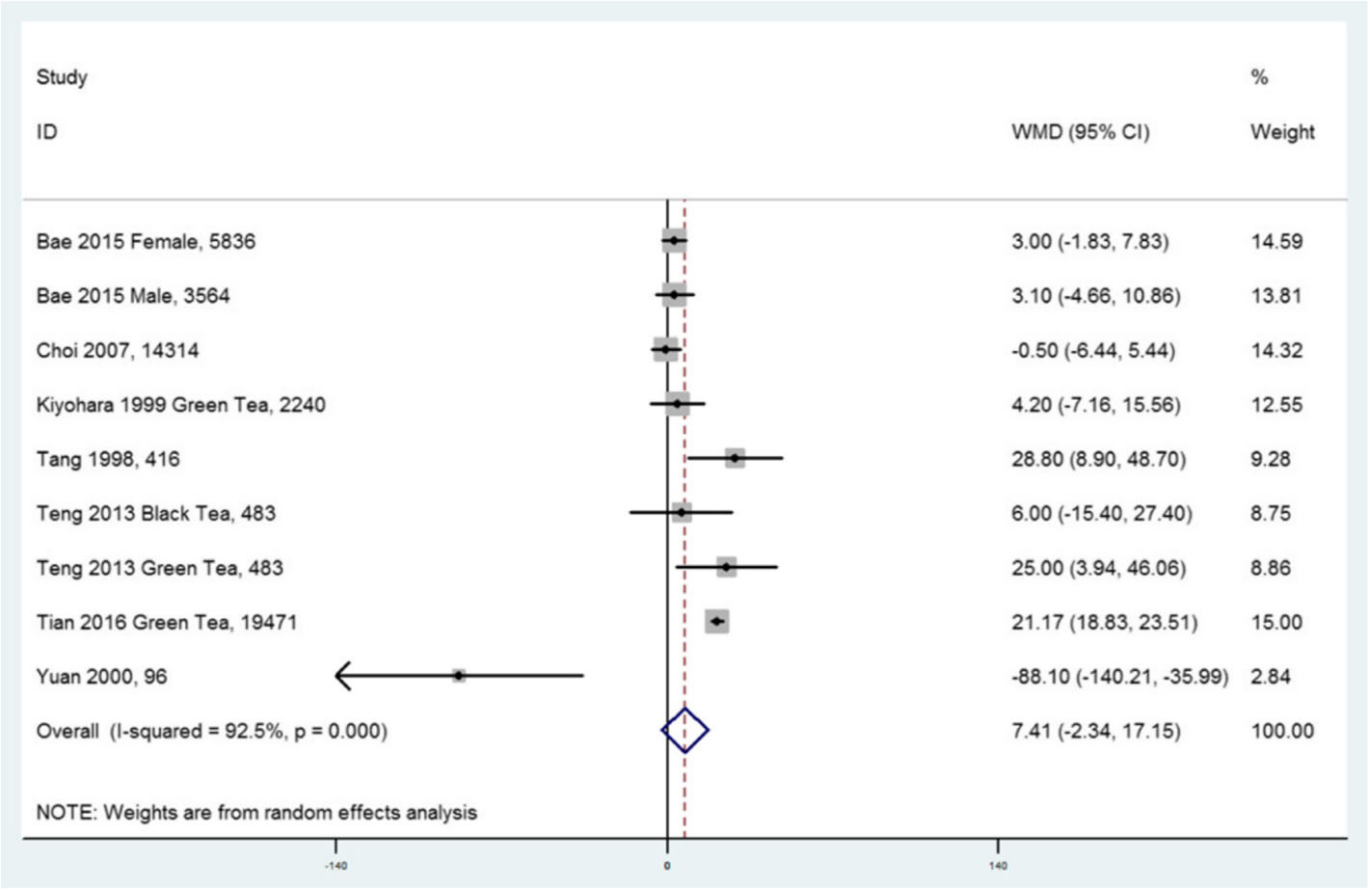
Forest plot of meta-analysis: WMD of SUA concentration between the highest and the lowest tea intake category

**Fig. 3:**
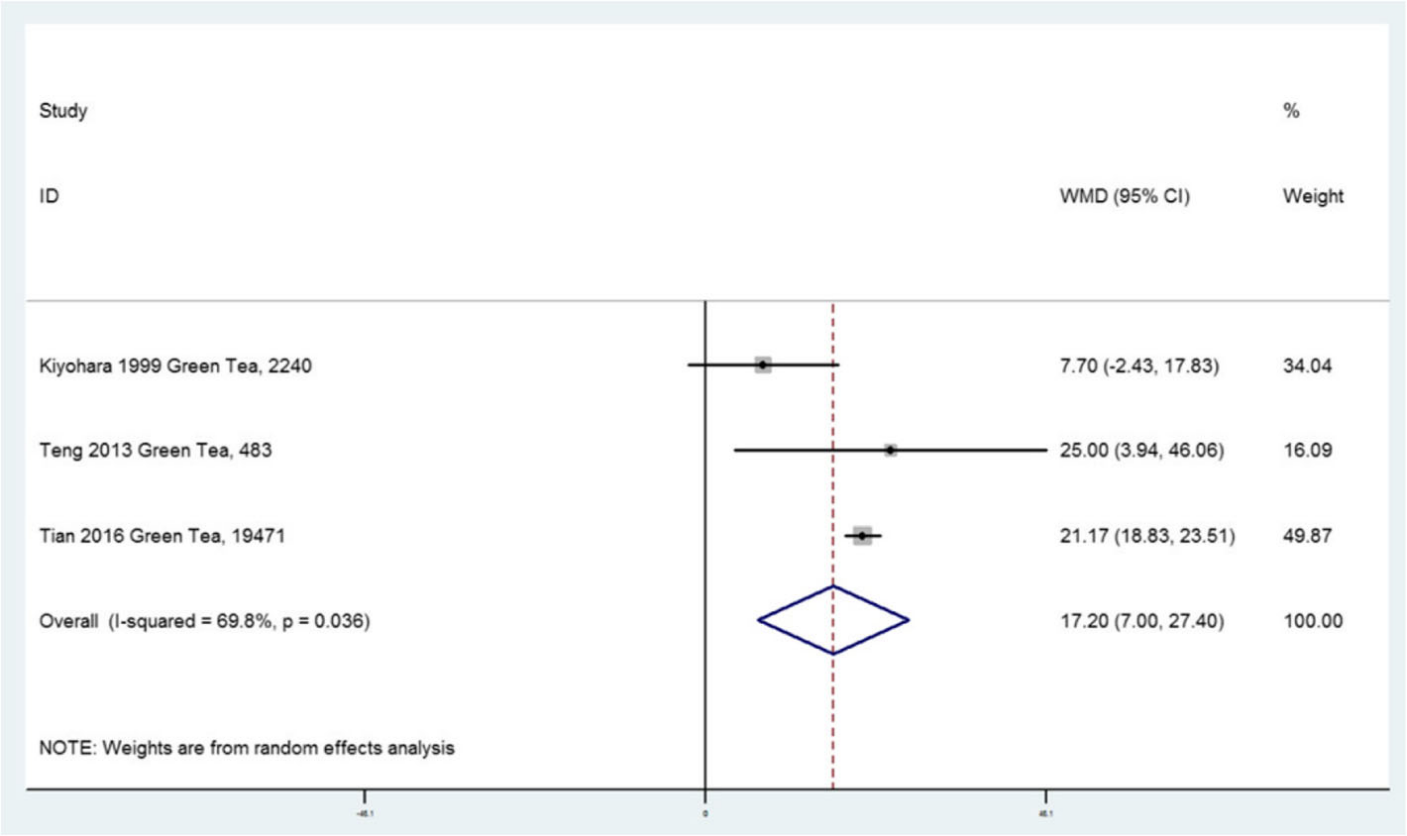
Forest plot of meta-analysis: WMD of SUA concentration between the highest and the lowest green tea intake category

Seven studies including five cross-sectional, one cohort and one case–control studies, reported the SUA concentration in different tea intake categories [32, 34, 36, 37, 44–46]. They originated from USA, China (two studies), Taiwan, Japan, Korea and Singapore. At the level of study setting, six population-based and one hospital-based studies were included. Since Tian only provides baseline and five year follow-up SUA level in tea consumers and non-tea consumers, the baseline data was extracted into this meta-analysis [46]. The combined WMD suggested that there was no significant difference in SUA between the highest and the lowest tea intake category (WMD = 7.41 μmol/L, 95%CI: −2.34 to 17.15; *P =* 0.136) (Fig. 2). A substantial level of heterogeneity was observed among studies (*P <* 0.001, I^2^ = 93%). No evidence of publication bias was observed among the included studies according to the Begg rank-correlation test (*P =* 0.917). Since Yuan’s study [45] has a relatively small weighting and seems like a considerable outlier (two thirds of participants were HU or gout patients), a sensitivity analysis was conducted. The results showed that there was a moderately increase in the SUA level for the highest versus the lowest tea intake category (WMD = 10.08 μmol/L, 95%CI: 0.79 to 19.38; *P =* 0.033). A substantial level of heterogeneity was observed among studies (*P <* 0.001, I^2^ = 92%). No evidence of publication bias was observed among the included studies according to the Begg rank-correlation test (*P =* 0.536). Three studies were included in a subgroup analysis for green tea. The combined WMD suggested that green tea consumption was positively associated with the SUA level (WMD = 17.20 μmol/L, 95%CI: 7.00 to 27.40; *P =* 0.01) (Fig. 3). A substantial level of heterogeneity was observed among studies (*P =* 0.036, I^2^ = 70%). No evidence of publication bias was observed among the included studies according to the Begg rank-correlation test (*P =* 1.00). It is necessary to emphasize that there were four studies with inappropriate data for meta-analysis. Chang [47], Curb [48] and Chatzistamatiou [49] only reported the correlation coefficient between tea and the SUA level. In addition, the relative data was not showed in Haldar [50]. Chang and Haldar found that tea consumption was positively associated with the SUA concentration; while Curb and Chatzistamatiou reported a negative relationship between the two.

### Odds ratio of HU for the highest versus the lowest tea intake category

**Fig. 4:**
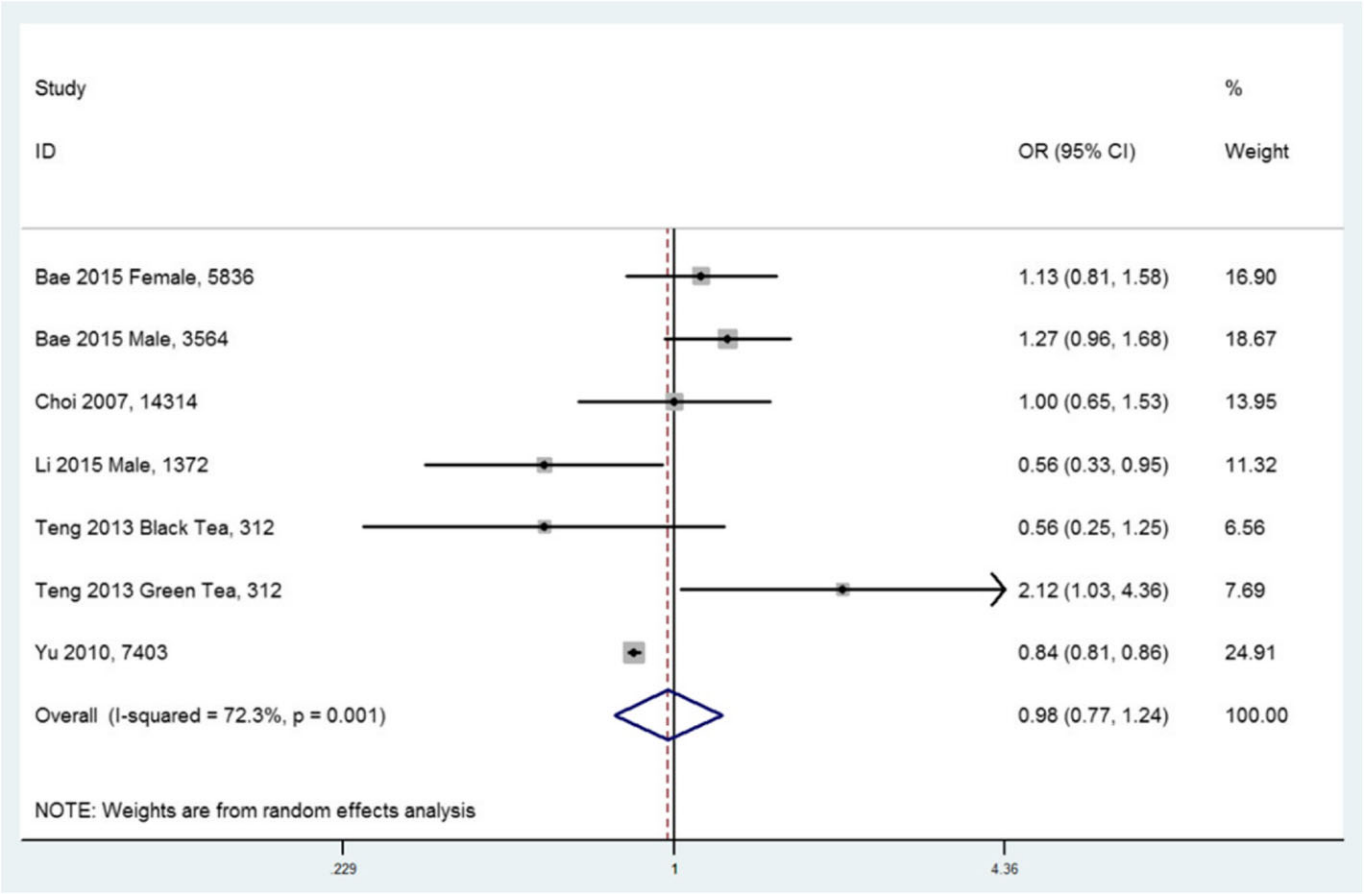
Forest plot of meta-analysis: Overall multi-variable adjusted OR of HU for the highest versus the lowest category of tea consumption

Five cross-sectional studies reported the OR for the prevalence of HU [34, 36–38, 51]. They were all community population-based studies which originated from USA, China (two studies), Korea and Singapore. The overall multi-variable adjusted OR for the highest versus the lowest category of tea intake showed no significant difference (OR = 0.98, 95%CI: 0.77 to 1.24, *P =* 0.839) (Fig. 4). A substantial level of heterogeneity was observed among studies (*P =* 0.001, I^2^ = 72%). No evidence of publication bias was observed among the included studies according to the Begg rank-correlation test (*P =* 1.00).

### Relative risk of gout for the highest versus the lowest tea intake category

Only two prospective cohort studies reported the RR for the risk of gout [33, 35]. They were both community population-based studies which originated from USA. Their results showed that tea consumption does not seem to be associated with the risk of gout in males (RR = 0.82, 95%CI: 0.38 to 1.75) and females (RR = 1.55, 95%CI: 0.98 to 2.47), respectively.

## Discussion

A total of fifteen studies were included in this systematic review and meta-analysis. Nine studies were retrieved to examine the associations of tea consumption with the SUA level and HU in meta-analysis. The quantitative synthesis of these observational studies showed that there was no significant relationship between tea consumption and the SUA level or HU. However, green tea consumption might be positively associated with the SUA level. In addition, two prospective cohort studies showed that tea consumption was not associated with the risk of gout.

Recently, a meta-analysis including five randomized controlled trials aimed to explore the influence of tea or tea extracts on the SUA level [52]. Unfortunately, due to the limited number of included studies and the lack of data on bioavailability (bioavailability is the proportion of the dose of a drug that reaches the systemic circulation intact after administration by a route other than intravenous), it failed to clarify any effective influence. However tea extracts could increase the SUA level in normal subjects but decrease that in HU patients [52]. This interesting phenomenon could be due to the dual effects of polyphenols on the SUA level. Polyphenols could decrease the production and increase the excretion of uric acid (UA) [53–55], but may also prevent oxidation [33–35] (UA is an antioxidant). Further studies are therefore needed to elaborate these issues.

Although green tea and black tea are both derived from Camellia sinensis, they are processed differently. In the manufacturing process of green tea, fresh tea leaves are steamed or heated immediately after harvest and result in minimal oxidation of polyphenols. Therefore, the major polyphenols in green tea are epigallocatechin gallate (EGCG). On the contrary, in the manufacturing process of black tea, tea leaves are dried and crushed to enhance oxidation, which generates more kinds of polyphenols (e.g. theaflavins and thearubigens) [52]. Some experimental studies reported the effect of green tea extracts in decreasing the SUA level in rat or mice. Jung [29] and Meki [30] showed that green tea extracts could reduce the SUA level in metabolic syndrome and rheumatoid arthritis rat models. In addition, Chen [31] further confirmed that green tea polyphenols could lower the SUA level in mice with HU by decreasing UA production and enhancing UA excretion. Therefore, green tea consumption might be negatively associated with the SUA level, HU and the risk of gout. Although the combined WMD suggested that there was no significant relationship between tea consumption and the SUA level, the majority of WMD values actually showed an increase in SUA level for tea group. Moreover, a sensitivity analysis excluding Yuan’s study [45], showed that tea consumption was even moderately positively associated with the SUA level. Therefore, we speculate some varieties of tea might increase the SUA level. Since only one study specified the varieties of tea (green tea, black tea) [36] and two studies investigated the green tea specially [32, 46], a subgroup analysis (three studies) for green tea was conducted. Surprisingly, their results showed that green tea consumption was positively associated with the SUA level. For this obvious contradiction between experimental and epidemiological studies, several speculations were listed as follow. To begin with, the reliability of this results might be weaken since only three studies were included for subgroup analysis. Besides, the green tea extracts or polyphenols might decrease the SUA level in animal model rather than in human beings. Furthermore, the components in green tea were complicated and some neglected substance might increase the SUA level, which ran counter to the effect of polyphenols. Finally, polyphenols might has a dual effect on the SUA level which depends on the SUA level itself. In another word, tea extracts could increase the SUA level in normal subjects but decrease that in HU patients [52], which might partly account for the difference in WMD which occurred as a result of the sensitivity analysis. Nevertheless, we did not find any associations of tea consumption with HU or the risk of gout. More well-designed studies with classification of different tea varieties are needed.

The strengths of the present systematic review and meta-analysis are mainly reflected from the following aspects. First, this is the first systematic review and meta-analysis aiming at the associations of tea consumption with the SUA level, HU and the risk of gout based on the most comprehensive literature search to date. Second, the included studies were analyzed based on adjusted results and large samples. Third, this study reveals the potential contradiction between experimental and epidemiological studies for green tea. Limitations of the present study should also be acknowledged. Firstly, the substantial level of heterogeneity among various studies might have distorted the results of this meta-analysis. Secondly, due to the limitation of relevant literature, only a few studies were qualified for this meta-analysis. Thirdly, it is difficult to evaluate the classification of tea intake. Tea consumption was mostly assessed by the number of cups of daily intake, but the concentration of each variety of tea and the cup size could vary greatly among individuals. Fourthly, the definitions of outcome and the selection of adjusted factors were not uniform. Last but not the least, since only a small number of studies specified the varieties of tea, some issues could not be addressed. These limitations might weaken the strength of this study.

## Conclusion

In conclusion, the current evidences suggest that tea consumption does not seem to be associated with the SUA level, HU and the risk of gout. However, due to the limited number of studies, green tea consumption might be positively associated with the SUA level. More well-designed prospective cohort studies, which classify the varieties of tea, are needed to elaborate these issues further.

## Additional files

Additional file 1: Search string. (DOCX 89 kb)
Additional file 2: Excluded articles. (DOCX 14 kb)
Additional file 3: Table S1. The methodological quality of cross-sectional studies in accordance with the Newcastle-Ottawa Scale (NOS). **Table S2**. The methodological quality of cohort studies in accordance with the Newcastle-Ottawa Scale (NOS). **Table S3**. The methodological quality of case–control studies in accordance with the Newcastle-Ottawa Scale (NOS). (DOCX 19 kb)

## Abbreviations

CI: Confidence interval
ECCG: Epigallocatechin gallate
HU: Hyperuricemia
OR: Odds ratio
RR: Relative risk
SUA: Serum uric acid
UA: Uric acid
WMD: Weighted mean difference
