# Supplementary material for: Is tea consumption associated with the serum uric acid level, hyperuricemia or the risk of gout? A systematic review and meta-analysis
Source: BMC Musculoskelet Disord. 2017 Feb 28;18:95. doi: 10.1186/s12891-017-1456-x (PMC5331744; doi:10.1186/s12891-017-1456-x)
Supplement: Additional file 1: — Search string. (DOCX 89 kb) [file 12891_2017_1456_MOESM1_ESM.docx]

**Irrelevant articles:**

[1]. Nag Chaudhuri, A.K., et al., Anti-inflammatory activity of Indian black tea (Sikkim variety). Pharmacological Research, 2005. 51(2): p. 169-175.

[2]. Henning, S.M., et al., Bioavailability and antioxidant effect of epigallocatechin gallate administered in purified form versus as green tea extract in healthy individuals. J Nutr Biochem, 2005. 16(10): p. 610-6.

[3]. Azam, S., et al., Antioxidant and prooxidant properties of caffeine, theobromine and xanthine. Med Sci Monit, 2003. 9(9): p. BR325-30.

[4]. Kitagawa, S., et al., Effectiveness of OS-1 for Water and Electrolyte Supplementation in Elderly Dehydrated Patients - Multicenter Clinical Study Using Commercially Available Mineral Water as a Control Solution. Japanese Pharmacology and Therapeutics, 2003. 31(10): p. 855-868.

[5]. Schlesier, K., et al., Assessment of antioxidant activity by using different in vitro methods. Free Radic Res, 2002. 36(2): p. 177-87.

[6]. Chen, G., et al., Separation of six purine bases by capillary electrophoresis with electrochemical detection. Analytica Chimica Acta, 2002. 457(2): p. 225-233.

[7]. Zheng, X.Q., et al., Theacrine (1,3,7,9-tetramethyluric acid) synthesis in leaves of a Chinese tea, kucha (Camellia assamica var. kucha). Phytochemistry, 2002. 60(2): p. 129-34.

[8]. Premgamone, A., et al., A long-term study on the efficacy of a herbal plant, Orthosiphon grandiflorus, and sodium potassium citrate in renal calculi treatment. Southeast Asian J Trop Med Public Health, 2001. 32(3): p. 654-60.

[9]. Van Amelsvoort, J.M., et al., Plasma concentrations of individual tea catechins after a single oral dose in humans. Xenobiotica, 2001. 31(12): p. 891-901.

[10]. Kessler, T. and A. Hesse, Cross-over study of the influence of bicarbonate-rich mineral water on urinary composition in comparison with sodium potassium citrate in healthy male subjects. Br J Nutr, 2000. 84(6): p. 865-71.

[11]. Lin, J.K., et al., Inhibition of xanthine oxidase and suppression of intracellular reactive oxygen species in HL-60 cells by theaflavin-3,3'-digallate, (-)-epigallocatechin-3-gallate, and propyl gallate. J Agric Food Chem, 2000. 48(7): p. 2736-43.

[12]. Colussi, G., et al., Medical prevention and treatment of urinary stones. Journal of nephrology, 2000. 13 Suppl 3: p. S65-70.

[13]. Stickel, F. and H.K. Seitz, The efficacy and safety of comfrey. Public Health Nutr, 2000. 3(4A): p. 501-8.

[14]. Lean, M.E., et al., Dietary flavonols protect diabetic human lymphocytes against oxidative damage to DNA. Diabetes, 1999. 48(1): p. 176-81.

[15]. Lean, M.E.J., et al., Dietary flavonols protect diabetic human lymphocytes against oxidative damage to DNA. Diabetes, 1999. 48(1): p. 176-181.

[16]. Ghiselli, A. and M. Serafini, Diet and total plasma antioxidant capacity in humans. Medecine Biologie Environnement, 1998. 26(2): p. 171-175.

[17]. Pietta, P. and P. Simonetti, Dietary flavonoids and interaction with endogenous antioxidants. Biochemistry and Molecular Biology International, 1998. 44(5): p. 1069-1074.

[18]. Koscielny, J., et al., Fagorutin buckwheat herb tea in chronic venous insufficiency. Zeitschrift fur Phytotherapie, 1996. 17(3): p. 147-159.

[19]. Reed, T., et al., Correlations of alcohol consumption with related covariates and heritability estimates in older adult males over a 14- to 18-year period: the NHLBI Twin Study. Alcohol Clin Exp Res, 1994. 18(3): p. 702-10.

[20]. Brokl, O.H., E.J. Braun and W.H. Dantzler, Transport of PAH, urate, TEA, and fluid by isolated perfused and nonperfused avian renal proximal tubules. Am J Physiol, 1994. 266(4 Pt 2): p. R1085-94.

[21]. Hesse, A., et al., The influence of dietary factors on the risk of urinary stone formation. Scanning Microsc, 1993. 7(3): p. 1119-27; discussion 1127-8.

[22]. Cloutier, M.M. and L. Guernsey, p-Aminohippurate transport in airways: competitive inhibition. Am J Physiol, 1992. 262(5 Pt 1): p. L555-9.

[23]. Tang, B.K., et al., Caffeine as a metabolic probe: validation of its use for acetylator phenotyping. Clin Pharmacol Ther, 1991. 49(6): p. 648-57.

[24]. Nirdnoy, M. and V. Muangman, Effects of Folia orthosiphonis on urinary stone promoters and inhibitors. J Med Assoc Thai, 1991. 74(6): p. 318-21.

[25]. Noorwali, A.A., et al., Tea consumption as a possible risk factor in urolithiasis: A preliminary report. Annals of Saudi Medicine, 1988. 8(2): p. 108-112.

[26]. Klatsky, A.L., G.D. Friedman and M.A. Armstrong, The relationships between alcoholic beverage use and other traits to blood pressure: a new Kaiser Permanente study. Circulation, 1986. 73(4): p. 628-36.

[27]. Tiktinsky, O.L. and A. Bablumyan Yu., The therapeutic effect of Java tea and Equisetum arvense in patients with uratic diathesis. Urologiya i Nefrologiya, 1983. 48(1): p. 47-50.

[28]. Tiktinskii, O.L. and I.A. Bablumian, Therapeutic action of Java tea and field horsetail in uric acid diathesis. Urologiia i nefrologiia, 1983(1): p. 47-50.

[29]. Bierer, D., T. Wang and A.J. Quebbemann, Effect of levodopa (L-DOPA) and its metabolites on the renal tubular excretory transport of 14C-uric acid (UA), p-amino-hippuric acid (PAH) and tetraethylammonium (TEA). Federation Proceedings, 1979. 38(3 I): p. No. 3275.

[30]. Kihlman, B.A., 1,3,7,9-tetramethyluric acid - a chromosome-damaging agent occurring as a natural metabolite in certain caffeine-producing plants. Mutation Research, 1977. 39(3-4): p. 297-315.

[31]. Campdelacreu, J., Parkinson disease and Alzheimer disease: environmental risk factors. Neurologia, 2012.

[32]. Crew, K.D., et al., Phase IB randomized, double-blinded, placebo-controlled, dose escalation study of polyphenon E in women with hormone receptor-negative breast cancer. Cancer Prevention Research, 2012. 5(9): p. 1144-1154.

[33]. Sluijs, I., et al., Plasma uric acid and risk of type 2 diabetes: The influence of a uric acid related dietary pattern and metabolic risk factors. Circulation, 2012. 125(10).

[34]. Alyami, F.A. and D.M. Rabah, Effect of drinking parsley leaf tea on urinary composition and urinary stones' risk factors. Saudi J Kidney Dis Transpl, 2011. 22(3): p. 511-4.

[35]. Muhammad, H., et al., Evaluation of the genotoxicity of Orthosiphon stamineus aqueous extract. J Ethnopharmacol, 2011. 133(2): p. 647-53.

[36]. Ladwa, R., A. Gruber and R. Smith, Exenatide: A cure for fatty liver and metabolic syndrome? Journal of Diabetes, 2011. 3: p. 199.

[37]. Padmini, E. and M. Usha Rani, Lipid profile alterations and oxidative stress in patients with preeclampsia: Role of black tea extract on disease management. Turkish Journal of Medical Sciences, 2011. 41(5): p. 761-768.

[38]. Rieder, A. and C.N. Negreiros, Plant known as Sarsaparilla [Herreria sarsaparilla Mart. [Herreriaceae] and its medicinal use in southwestern Mato Grosso, Brazil. Planta Medica, 2011. 77(12).

[39]. Neogi, T., et al., Short-term effects of caffeinated beverage intake on risk of recurrent gout attacks. Arthritis and Rheumatism, 2010. 62: p. 1362.

[40]. Brandabur, M., Complementary drug options for PD. Movement Disorders, 2010. 25: p. S587.

[41]. Tanner, C., Epidemiology of PD. Movement Disorders, 2010. 25: p. S576.

[42]. Al-Attar, A.M. and T.A. Zari, Influences of crude extract of tea leaves, Camellia sinensis, on streptozotocin diabetic male albino mice. Saudi J Biol Sci, 2010. 17(4): p. 295-301.

[43]. Hu, F.B. and V.S. Malik, Sugar-sweetened beverages and risk of obesity and type 2 diabetes: Epidemiologic evidence. Physiology and Behavior, 2010. 100(1): p. 47-54.

[44]. Li, L., et al., Supplementation with lutein or lutein plus green tea extracts does not change oxidative stress in adequately nourished older adults. Journal of Nutritional Biochemistry, 2010. 21(6): p. 544-549.

[45]. Poncelet, J., et al., The effect of early experience on odor perception in humans: Psychological and physiological correlates. Behavioural Brain Research, 2010. 208(2): p. 458-465.

[46]. Chen, S.Y. and S.T. Tsai, The epidemiology of Parkinson's disease. Tzu Chi Medical Journal, 2010. 22(2): p. 73-81.

[47]. Peng, X.E., et al., [A hospital-based case-control study on influencing factors of nonalcoholic fatty liver disease]. Zhonghua gan zang bing za zhi = Zhonghua ganzangbing zazhi = Chinese journal of hepatology, 2009. 17(7): p. 535-539.

[48]. Fatiha, L., et al., Correlations between the composition of Moroccan urinary stones and the risk factors (food habit). Pakistan Journal of Nutrition, 2009. 8(7): p. 977-982.

[49]. Pereira, Z.V., et al., Medicinal plants used by Ponta Porã community, Mato Grosso do Sul State. Acta Scientiarum - Biological Sciences, 2009. 31(3): p. 293-299.

[50]. Lu, Z., et al., Serum uric acid level in primary hypertension among Chinese nonagenarians/centenarians. J Hum Hypertens, 2009. 23(2): p. 113-21.

[51]. Chen, Y., et al., Significant differences of some physiological parameters in humans and pigs. Xenotransplantation, 2009. 16(5): p. 437.

[52]. Nguyen, S., et al., Sugar-Sweetened Beverages, Serum Uric Acid, and Blood Pressure in Adolescents. Journal of Pediatrics, 2009. 154(6): p. 807-813.

[53]. Bugdayci, G., Y. Balaban and O. Sahin, Causes of hypouricemia among outpatients. Laboratory Medicine, 2008. 39(9): p. 550-552.

[54]. Elbaz, A. and F. Moisan, Update in the epidemiology of Parkinson's disease. Curr Opin Neurol, 2008. 21(4): p. 454-60.

[55]. Seidel, C., et al., Influence of prebiotics and antioxidants in bread on the immune system, antioxidative status and antioxidative capacity in male smokers and non-smokers. Br J Nutr, 2007. 97(2): p. 349-56.

[56]. Padmini, E. and B. Vijaya Geetha, In-vitro studies on the effect of tea extracts on the small dense IDL oxidation in preeclampsia. Biomedicine, 2007. 27(4): p. 168-172.

[57]. Polagruto, J.A., et al., Platelet reactivity in male smokers following the acute consumption of a flavanol-rich grapeseed extract. J Med Food, 2007. 10(4): p. 725-30.

[58]. Shpigun, L.K., et al., Flow injection potentiometric determination of total antioxidant activity of plant extracts. Anal Chim Acta, 2006. 573-574: p. 419-26.

[59]. Yang, Q.P., et al., Influence of obesity on heart rate variability. Chinese Journal of Clinical Rehabilitation, 2006. 10(48): p. 25-27.

[60]. Rabovsky, A., J. Cuomo and N. Eich, Measurement of plasma antioxidant reserve after supplementation with various antioxidants in healthy subjects. Clin Chim Acta, 2006. 371(1-2): p. 55-60.

[61]. Elsayed, A.S.I., Alterations in serum biochemical parameters in response to gasoline inhalation and the protective effects of green tea and curcumin. Pakistan Journal of Nutrition, 2016. 15(1): p. 15-22.

[62]. de Moraes, I.V.M., et al., UPLC–QTOF–MS and NMR analyses of graviola (Annona muricata) leaves. Brazilian Journal of Pharmacognosy, 2016. 26(2): p. 174-179.

[63]. Zarin, F., T. Kazemi and P. Vakili, A review of kidney stone and its risk factors along with diagnostic methods. Research Journal of Pharmaceutical, Biological and Chemical Sciences, 2015. 6(2): p. 920-925.

[64]. Syed, A. and N. Sheikh, Age dependant counter impact of fat reducing agents on renal function test of diet induced non alcoholic fatty liver disease in Rattus norvegicus. Hepatology International, 2015. 9(1): p. S370.

[65]. Waheed, A., et al., Beverages: Alternative medicine and health benefits. International Journal of Pharmacy and Technology, 2015. 6(4): p. 7568-7586.

[66]. Mangueira, L.F.B., et al., Clinical safety evaluation of a tea containing Cissampelos sympodialis in healthy volunteers. Brazilian Journal of Pharmacognosy, 2015. 25(5): p. 491-498.

[67]. Simon, P.W., C. Pao and M.E. Rybak, Determination of urine caffeine and caffeine metabolites by use of polarity-switching LC-MS/MS. Clinical Chemistry, 2015. 61(10): p. S89.

[68]. Lee, C.Y., et al., Efficacy and safety of herbal medicine yun-cai tea in the treatment of hyperlipidemia: A double-blind placebo-controlled clinical trial. Chinese Journal of Integrative Medicine, 2015. 21(8): p. 587-593.

[69]. Stojkovic, D., et al., Ethnopharmacological uses of Sempervivum tectorum L. in southern Serbia: Scientific confirmation for the use against otitis linked bacteria. J Ethnopharmacol, 2015. 176: p. 297-304.

[70]. Peluso, I., M. Palmery and A. Vitalone, Green Tea and Bbone Marrow Transplantation: From Antioxidant Activity to Enzymatic and Multidrug-resistance Modulation. Crit Rev Food Sci Nutr, 2015: p. 0.

[71]. Liu, F., et al., Prevalence of isolated diastolic hypertension and associated risk factors among different ethnicity groups in Xinjiang, China. PLoS ONE, 2015. 10(12).

[72]. Orhan, N., et al., Preventive treatment of calcium oxalate crystal deposition with immortal flowers. J Ethnopharmacol, 2015. 163: p. 60-7.

[73]. Chiang, W.F., et al., Rhabdomyolysis induced by excessive coffee drinking. Human and Experimental Toxicology, 2015. 33(8): p. 878-881.

[74]. Erol, S., et al., An interesting newborn case of fructose 1-6 diphosphatase deficiency triggered after thyme juice ingestion. Clinical Laboratory, 2014. 60(1): p. 151-153.

[75]. Laghari, M., et al., Evaluation of dietary and biochemical risk factors involved in the pathogenesis of bladder stones in children of below ten years age at Hyderabad Sindh. Medical Forum Monthly, 2014. 25(2): p. 88-91.

[76]. Miglio, C., et al., Fruit juice drinks prevent endogenous antioxidant response to high-fat meal ingestion. Br J Nutr, 2014. 111(2): p. 294-300.

[77]. Campdelacreu, J., Parkinson disease and Alzheimer disease: Environmental risk factors. Neurologia, 2014. 29(9): p. 541-549.

[78]. Hens, K., et al., Sigma metrics used to assess analytical quality of clinical chemistry assays: Importance of the allowable total error (TEa) target. Clinical Chemistry and Laboratory Medicine, 2014. 52(7): p. 973-980.

[79]. Kang, F.F., et al., Six sigma metric analysis for performance of four analytes with fresh frozen serum. Clinical Chemistry and Laboratory Medicine, 2014. 52: p. S1589.

[80]. Peker, S.A., et al., The calculation of the total error for the parameters studied in routine and emergency biochemistry laboratory. Turkish Journal of Biochemistry, 2014. 39: p. 78.

[81]. Rigaud, D., et al., Triggers of bulimia and compulsion attacks: Validation of the "start" questionnaire. Encephale, 2014. 40(4): p. 323-329.

[82]. Rohdiana, D., et al., Xanthine oxidase inhibitory and immunomodulatory activities of fifteen grades Indonesia orthodox black tea. International Journal of Pharmacy and Pharmaceutical Sciences, 2014. 6(5): p. 39-42.

[83]. Lin, W.T., et al., Effects of taiwan style fructose-rich beverages consumption on insulin resistance in adolescents. American Journal of Epidemiology, 2013. 177: p. S93.

[84]. Himani, B., et al., Misai kuching: A glimpse of maestro. International Journal of Pharmaceutical Sciences Review and Research, 2013. 22(2): p. 55-59.

[85]. Zheng, J.S., et al., Postprandial effects of two Chinese liquors on selected cardiovascular disease risk factors in young men. Acta Physiol Hung, 2013. 100(3): p. 302-11.

[86]. Guo, R., et al., Prevalence, awareness, treatment, control and associated risk factors of hypertension among middle-aged population of China: A multiplecenter cardiovascular epidemiological study, 2009-2010. Cardiology (Switzerland), 2013. 126: p. 14.

[87]. Chin, P.C., et al., Relationship between central obesity and erosive esophagitis in a non-obese taiwanese population. Obesity Facts, 2013. 6: p. 211.

[88]. Rivard, C., et al., Sack and sugar, and the aetiology of gout in England between 1650 and 1900. Rheumatology (Oxford), 2013. 52(3): p. 421-6.

[89]. Cloetens, L., J. Panee and B. Åkesson, The antioxidant capacity of milk - The application of different methods in vitro and in vivo. Cellular and Molecular Biology, 2013. 59(1): p. 43-57.

[90]. Cloetens, L., J. Panee and B. Akesson, The antioxidant capacity of milk--the application of different methods in vitro and in vivo. Cell Mol Biol (Noisy-le-grand), 2013. 59(1): p. 43-57.

[91]. Chung, K.T., The etiology of bladder cancer and its prevention. Journal of Cancer Science and Therapy, 2013. 5(10): p. 346-361.

[92]. Watanabe, E., et al., Traditional Japanese “Maccha” type green tea (Camellia sinensis (L.) Kuntze) from Uji for metabolic syndrome therapy: An open-label clinical pilot study. Planta Medica, 2013. 79(13).

[93]. Lin, W.T., et al., Effects of taiwan style fructose-rich beverages intake on serum uric acid and body mass index in adolescents. American Journal of Epidemiology, 2012. 175: p. S21.

[94]. Zheng, J.S., et al., Effects of Chinese liquors on cardiovascular disease risk factors in healthy young humans. ScientificWorldJournal, 2012. 2012: p. 372143.

[95]. Anderson, R.F., et al., Fast chemical repair of free radical damage to DNA by the constituents and metabolites of beverages. Free Radical Biology and Medicine, 2012. 53: p. S192.

[96]. Noyce, A.J., et al., Meta-analysis of early nonmotor features and risk factors for Parkinson disease. Ann Neurol, 2012. 72(6): p. 893-901.

[97]. Agarwal, A. and M. Bansal, Obstructive jaundice due to hodgkin lymphoma: A rare entity. American Journal of Gastroenterology, 2012. 107: p. S445.

[98]. Shi, Y. and G. Williamson, Quercetin lowers plasma uric acid in pre-hyperuricaemic males: A randomised, double-blinded, placebo-controlled, cross-over trial. British Journal of Nutrition, 2016. 115(5): p. 800-806.

[99]. Ekpenyong, C.E., N.E. Daniel and A.B. Antai, Effect of lemongrass tea consumption on estimated glomerular filtration rate and creatinine clearance rate. J Ren Nutr, 2015. 25(1): p. 57-66.

[100]. Jowko, E., et al., The effect of green tea extract supplementation on exercise-induced oxidative stress parameters in male sprinters. Eur J Nutr, 2015. 54(5): p. 783-91.

[101]. Koutelidakis, A.E., et al., Effect of green tea on postprandial antioxidant capacity, serum lipids, C-reactive protein and glucose levels in patients with coronary artery disease. Eur J Nutr, 2014. 53(2): p. 479-86.

[102]. Jatuworapruk, K., et al., Effects of green tea extract on serum uric acid and urate clearance in healthy individuals. J Clin Rheumatol, 2014. 20(6): p. 310-3.

[103]. Boaventura, B.C., et al., Association of mate tea (Ilex paraguariensis) intake and dietary intervention and effects on oxidative stress biomarkers of dyslipidemic subjects. Nutrition, 2012. 28(6): p. 657-64.

[104]. Jowko, E., et al., Effect of a single dose of green tea polyphenols on the blood markers of exercise-induced oxidative stress in soccer players. Int J Sport Nutr Exerc Metab, 2012. 22(6): p. 486-96.

[105]. Hsu, C.H., et al., Does supplementation with green tea extract improve insulin resistance in obese type 2 diabetics? A randomized, double-blind, and placebo-controlled clinical trial. Alternative Medicine Review, 2011. 16(2): p. 157-163.

[106]. Chen, T.S., et al., Efficacy of epigallocatechin-3-gallate and amla (Emblica officinalis) extract for the treatment of diabetic-uremic patients. Journal of Medicinal Food, 2011. 14(7-8): p. 718-723.

[107]. Sone, T., et al., Randomized controlled trial for an effect of catechin-enriched green tea consumption on adiponectin and cardiovascular disease risk factors. Food Nutr Res, 2011. 55.

[108]. Bahorun, T., et al., Black tea reduces uric acid and C-reactive protein levels in humans susceptible to cardiovascular diseases. Toxicology, 2010. 278(1): p. 68-74.

[109]. Muller, N., et al., Bolus ingestion of white and green tea increases the concentration of several flavan-3-ols in plasma, but does not affect markers of oxidative stress in healthy non-smokers. Mol Nutr Food Res, 2010. 54(11): p. 1636-45.

[110]. Villaño, D., et al., Unfermented and fermented rooibos teas (Aspalathus linearis) increase plasma total antioxidant capacity in healthy humans. Food Chemistry, 2010. 123(3): p. 679-683.

[111]. Panza, V.S., et al., Consumption of green tea favorably affects oxidative stress markers in weight-trained men. Nutrition, 2008. 24(5): p. 433-42.

[112]. Gomikawa, S., et al., Effect of ground green tea drinking for 2 weeks on the susceptibility of plasma and LDL to the oxidation ex vivo in healthy volunteers. Kobe J Med Sci, 2008. 54(1): p. E62-72.

[113]. Prasongwatana, V., et al., Uricosuric effect of Roselle (Hibiscus sabdariffa) in normal and renal-stone former subjects. J Ethnopharmacol, 2008. 117(3): p. 491-5.

[114]. Hsu, T.F., et al., Polyphenol-enriched oolong tea increases fecal lipid excretion. European Journal of Clinical Nutrition, 2006. 60(11): p. 1330-1336.

[115]. Natella, F., et al., Coffee drinking influences plasma antioxidant capacity in humans. J Agric Food Chem, 2002. 50(21): p. 6211-6.

[116]. Kimura, M., et al., The relation between single/double or repeated tea catechin ingestions and plasma antioxidant activity in humans. Eur J Clin Nutr, 2002. 56(12): p. 1186-93.

[117]. Princen, H.M., et al., No effect of consumption of green and black tea on plasma lipid and antioxidant levels and on LDL oxidation in smokers. Arterioscler Thromb Vasc Biol, 1998. 18(5): p. 833-41.

[118]. Princen, H.M.G., et al., No effect of consumption of green and black tea on plasma lipid and antioxidant levels and on LDL Oxidation in smokers. Arteriosclerosis, Thrombosis, and Vascular Biology, 1998. 18(5): p. 833-841.

[119]. Hesse, A., et al., Medicinal teas in the prophylaxis of urinary calculus. Effect of Solubitrat on the excretion of lithogenic and inhibitory substances. Munchener Medizinische Wochenschrift, 1981. 123(13): p. 521-524.

**Non-human Study**:

[1]. Kondo, M., et al., Xanthine oxidase inhibitory activity and hypouricemic effect of aspalathin from unfermented rooibos. J Food Sci, 2013. 78(12): p. H1935-9.

[2]. Radwan, R.R., et al., Protection by low-dose γ radiation on doxorubicin-induced nephropathy in rats pretreated with curcumin, green tea, garlic or l-carnitine. Bulletin of Faculty of Pharmacy, Cairo University, 2012. 50(2): p. 133-140.

[3]. Alorainy, M.S., Effect of some antioxidants on bio-indices in arthritis induced in rats. Basic and Clinical Pharmacology and Toxicology, 2011. 109: p. 35.

[4]. Yang, X., et al., Effects of herbal tea or extract on uric acid parameters in humans and rats. FASEB Journal, 2011. 25.

[5]. Vasconcelos, C.F.B., et al., Hypoglycaemic activity and molecular mechanisms of Caesalpinia ferrea Martius bark extract on streptozotocin-induced diabetes in Wistar rats. Journal of Ethnopharmacology, 2011. 137(3): p. 1533-1541.

[6]. Bouanani, S., et al., Pharmacological and toxicological effects of Paronychia argentea in experimental calcium oxalate nephrolithiasis in rats. Journal of Ethnopharmacology, 2010. 129(1): p. 38-45.

[7]. Lin, S.M., et al., Protective effect of green tea (-)-epigallocatechin-3-gallate against the monoamine oxidase B enzyme activity increase in adult rat brains. Nutrition, 2010. 26(11-12): p. 1195-1200.

[8]. Jin, X., et al., Dietary fats altered nephrotoxicity profile of methylmercury in rats. J Appl Toxicol, 2009. 29(2): p. 126-40.

[9]. Meki, A.R., E.A. Hamed and K.A. Ezam, Effect of green tea extract and vitamin C on oxidant or antioxidant status of rheumatoid arthritis rat model. Indian J Clin Biochem, 2009. 24(3): p. 280-7.

[10]. Chander, R., et al., Antioxidant and lipid lowering activities of Indian black tea. Indian J Clin Biochem, 2005. 20(1): p. 153-9.

[11]. Leena, P. and R. Balaraman, Effect of green tea extract on cisplatin induced oxidative damage on kidney and testes of rats. Ars Pharmaceutica, 2005. 46(1): p. 5-18.

[12]. Kim, J.W., et al., Effect of green tea on calcium oxalate stone formation and excretion in ethylene glycol-treated rats. Korean Journal of Urology, 2005. 46(3): p. 299-305.

[13]. Mohamadin, A.M., H.A. El-Beshbishy and M.A. El-Mahdy, Green tea extract attenuates cyclosporine A-induced oxidative stress in rats. Pharmacol Res, 2005. 51(1): p. 51-7.

[14]. Habu, Y., et al., Restored expression and activity of organic ion transporters rOAT1, rOAT3 and rOCT2 after hyperuricemia in the rat kidney. Biochem Pharmacol, 2005. 69(6): p. 993-9.

[15]. Zhang, X., et al., Relative contribution of OAT and OCT transporters to organic electrolyte transport in rabbit proximal tubule. Am J Physiol Renal Physiol, 2004. 287(5): p. F999-1010.

[16]. Habu, Y., et al., Decreased activity of basolateral organic ion transports in hyperuricemic rat kidney: roles of organic ion transporters, rOAT1, rOAT3 and rOCT2. Biochem Pharmacol, 2003. 66(6): p. 1107-14.

[17]. Habu, Y., et al., Decreased activity of basolateral organic ion transports in hyperuricemic rat kidney: Roles of organic ion transporters, rOAT1, rOAT3 and rOCT2. Biochemical Pharmacology, 2003. 66(6): p. 1107-1114.

[18]. Balasubramanian, T., Uric acid or 1-methyl uric acid in the urinary bladder increases serum glucose, insulin, true triglyceride, and total cholesterol levels in Wistar rats. ScientificWorldJournal, 2003. 3: p. 930-6.

[19]. Gomikawa, S. and Y. Ishikawa, Effects of catechins and ground green tea drinking on the susceptibility of plasma and LDL to the oxidation in vitro and ex vivo. Journal of Clinical Biochemistry and Nutrition, 2002. 32: p. 55-68.

[20]. Aucamp, J., et al., Inhibition of xanthine oxidase by catechins from tea (Camellia sinensis). Anticancer Res, 1997. 17(6D): p. 4381-5.

[21]. Nakajima, M., et al., Transport of papaverine in rat kidney cortical slices. J Pharmacobiodyn, 1984. 7(11): p. 830-5.

[22]. Albokhadaim, I., Effect of aqueous extract of green tea (Camellia sinensis) on hematology and oxidative stress biomarkers in rats intoxicated with carbon tetrachloride. Journal of Biological Sciences, 2016. 16(3): p. 49-57.

[23]. Jhang, J.J., C.C. Lu and G.C. Yen, Epigallocatechin gallate inhibits urate crystals-induced peritoneal inflammation in C57BL/6 mice. Mol Nutr Food Res, 2016.

[24]. Li, J., et al., Green tea extract provides extensive Nrf2-independent protection against lipid accumulation and NFkappaB pro- inflammatory responses during nonalcoholic steatohepatitis in mice fed a high-fat diet. Mol Nutr Food Res, 2016. 60(4): p. 858-70.

[25]. Li, S.B., et al., Differing chemical compositions of three teas may explain their different effects on acute blood pressure in spontaneously hypertensive rats. J Sci Food Agric, 2015. 95(6): p. 1236-42.

[26]. Wilson, R.D. and M.S. Islam, Effects of white mulberry (Morus alba) leaf tea investigated in a type 2 diabetes model of rats. Acta Pol Pharm, 2015. 72(1): p. 153-60.

[27]. Xie, H., et al., Epigallocatechin-3-gallate attenuates uric acid-induced inflammatory responses and oxidative stress by modulating notch pathway. Journal of Hypertension, 2015. 33: p. e23.

[28]. Chen, G., et al., Green tea polyphenols decreases uric acid level through xanthine oxidase and renal urate transporters in hyperuricemic mice. J Ethnopharmacol, 2015. 175: p. 14-20.

[29]. Turgut, C.D., et al., Impact of tannic acid on blood pressure, oxidative stress and urinary parameters in L-NNA-induced hypertensive rats. Cytotechnology, 2015. 67(1): p. 97-105.

[30]. Ait Hamadouche, N. and A. Hadi, The protective effect of green tea extract on lead induced oxidative and damage on rat kidney. International Journal of Pharma and Bio Sciences, 2015. 6(1): p. P97-P107.

[31]. Yun, S.W. and M.R. Kim, Antidiabetic effect of red ginseng extract on type I diabetes in mouse induced alloxan. Diabetes Research and Clinical Practice, 2014. 106: p. S244.

[32]. Acharyya, N., S. Chattopadhyay and S. Maiti, Chemoprevention against arsenic-induced mutagenic DNA breakage and apoptotic liver damage in rat via antioxidant and SOD1 upregulation by green tea (Camellia sinensis) which recovers broken DNA resulted from arsenic-H2O2 related in vitro oxidant stress. J Environ Sci Health C Environ Carcinog Ecotoxicol Rev, 2014. 32(4): p. 338-61.

[33]. Ferrari, F.C., et al., Effects of Pimenta pseudocaryophyllus ethanolic extracts on serum uric acid levels in oxonate-induced mice and xanthine oxidase activity in vitro and in mouse liver. Planta Medica, 2014. 80(16).

[34]. Thangapandiyan, S. and S. Miltonprabu, Epigallocatechin gallate supplementation protects against renal injury induced by fluoride intoxication in rats: Role of Nrf2/HO-1 signaling. Toxicology Reports, 2014. 1: p. 12-30.

[35]. Oliviero, F., et al., Epigallocatechin gallate suppresses monosodium urate crystal-induced inflammation in mice. Annals of the Rheumatic Diseases, 2014. 73.

[36]. Ramesh, K., S. Manohar and S. Rajeshkumar, Nephroprotective activity of ethanolic extract of Orthosiphon stamineus leaves on ethylene glycol induced urolithiasis in albino rats. International Journal of PharmTech Research, 2014. 6(1): p. 403-408.

[37]. Hussein, S.A., O.A. Ragab and M.A. El-Eshmawy, Protective effect of green tea extract on cyclosporine a: Induced nephrotoxicity in rats. Journal of Biological Sciences, 2014. 14(4): p. 248-257.

[38]. Elzoghby, R.R., et al., Protective role of vitamin C and green tea extract on malathion-induced hepatotoxicity and nephrotoxicity in rats. American Journal of Pharmacology and Toxicology, 2014. 9(3): p. 174-185.

[39]. Jung, M.H., et al., Effect of green tea extract microencapsulation on hypertriglyceridemia and cardiovascular tissues in high fructose-fed rats. Nutr Res Pract, 2013. 7(5): p. 366-72.

[40]. Turgut Coşan, D., et al., Impact of tannic acid on blood pressure, oxidative stress and urinary parameters in L-NNA-induced hypertensive rats. Cytotechnology, 2013: p. 1-9.

[41]. Chang, M., et al., Microencapsulated green tea extract supplementation ameliorated dyslipidemia and fat accumulation in a fructose fed rat model. Annals of Nutrition and Metabolism, 2013. 63: p. 1693.

[42]. Shuai, L., et al., The complex of tea functional components ameliorate renal injury induced by hyperuricemia in remnant kidney rats. Pediatric Nephrology, 2013. 28(8): p. 1353.

**Reviews, letters or cases:**

[1]. Li, Y., et al., Effects of tea or tea extract on metabolic profiles in patients with type 2 diabetes mellitus: A meta-analysis of ten randomized controlled trials. Diabetes/Metabolism Research and Reviews, 2016. 32(1): p. 2-10.

[2]. Peluso, I., et al., Camellia Sinensis in Asymptomatic Hyperuricaemia: A Meta-analysis of Tea or Tea Extract Effects on Uric Acid Levels. Crit Rev Food Sci Nutr, 2015: p. 0.

[3]. Towiwat, P. and Z.G. Li, The association of vitamin C, alcohol, coffee, tea, milk and yogurt with uric acid and gout. Int J Rheum Dis, 2015. 18(5): p. 495-501.

[4]. Venu Gopal, J., Morin Hydrate: Botanical origin, pharmacological activity and its applications: A mini-review. Pharmacognosy Journal, 2013. 5(3): p. 123-126.

[5]. Takashima, M., et al., Assessment of antioxidant capacity for scavenging free radicals in vitro: a rational basis and practical application. Free Radic Biol Med, 2012. 52(7): p. 1242-52.

[6]. Noyce, A.J., et al., Risk factors and early non-motor features for Parkinson's disease: A systematic review and meta-analysis. Movement Disorders, 2012. 27: p. S4.

[7]. Azzi, A., Antioxidants: They may be useful, but... In Vivo, 2011. 25(3): p. 472-473.

[8]. Ali, B.H., et al., Experimental gentamicin nephrotoxicity and agents that modify it: a mini-review of recent research. Basic Clin Pharmacol Toxicol, 2011. 109(4): p. 225-32.

[9]. Ali, B.H., et al., Experimental Gentamicin Nephrotoxicity and Agents that Modify it: A Mini-Review of Recent Research. Basic and Clinical Pharmacology and Toxicology, 2011. 109(4): p. 225-232.

[10]. Al-Obaidi, H. and M. Afzal, Methylxanthine content in hot drinks consumed in the State of Kuwait. Journal of Food, Agriculture and Environment, 2010. 8(1): p. 41-43.

[11]. Łuczaj, W. and E. Skrzydlewska, Antioxidative properties of black tea. Preventive Medicine, 2005. 40(6): p. 910-918.

[12]. Numabe, A., et al., A case of pseudo-Bartter's syndrome induced by long-term ingestion of furosemide delivered orally through health tea. Japanese Journal of Nephrology, 2003. 45(5): p. 457-463.

[13]. Davidson, M.H. and C.T. Geohas, Efficacy of over-the-counter nutritional supplements. Current Atherosclerosis Reports, 2003. 5(1): p. 15-21.

[14]. L'Allemain, G., [Multiple actions of EGCG, the main component of green tea]. Bull Cancer, 1999. 86(9): p. 721-4.

[15]. Pillai, S.P., et al., Antimutagenic/antioxidant activity of green tea components and related compounds. Journal of Environmental Pathology, Toxicology and Oncology, 1999. 18(3): p. 147-158.

[16]. Ito, E., A. Crozier and H. Ashihara, Theophylline metabolism in higher plants. Biochim Biophys Acta, 1997. 1336(2): p. 323-30.

**Full-text articles unaccessible:**

[1]. Tiktinskii, O.L. and I. Bablumian, [Therapeutic action of Java tea and field horsetail in uric acid diathesis]. Urol Nefrol (Mosk), 1983(1): p. 47-50.

[2]. DECAUX, F. and B. BOURSIER, [Coffee, tea and cocoa in hyperuricemic patients]. Gaz Med Fr, 1963. 70: p. 1465-70.
