# Supplementary material for: Is tea consumption associated with the serum uric acid level, hyperuricemia or the risk of gout? A systematic review and meta-analysis
Source: BMC Musculoskelet Disord. 2017 Feb 28;18:95. doi: 10.1186/s12891-017-1456-x (PMC5331744; doi:10.1186/s12891-017-1456-x)
Supplement: Additional file 2: — Excluded articles. (DOCX 14 kb) [file 12891_2017_1456_MOESM2_ESM.docx]

Pubmed (96 articles)

1. Urate*[tiab] or “uric acid”[tiab] or gout[tiab] or hyperuric*emia[tiab]
2. “uric acid”[mesh] or "Gout"[Mesh] or "Hyperuricemia"[Mesh]
3. 1 or 2
4. “tea”[tiab]
5. 3 and 4

Embase (178 articles)

1. ‘uric acid’/exp
2. ‘gout’/exp
3. ‘Hyperuricemia’/exp
4. urate* or gout or hyperuric*emia:ti,ab
5. ‘tea’:ti,ab
6. or/1-4
7. 5 and 6

| Pubmed | Embase | Total |
| --- | --- | --- |
| 96 | 178 | 274 |
