# Supplementary material for: Is tea consumption associated with the serum uric acid level, hyperuricemia or the risk of gout? A systematic review and meta-analysis
Source: BMC Musculoskelet Disord. 2017 Feb 28;18:95. doi: 10.1186/s12891-017-1456-x (PMC5331744; doi:10.1186/s12891-017-1456-x)
Supplement: Additional file 3: Table S1. — The methodological quality of cross-sectional studies in accordance with the Newcastle-Ottawa Scale (NOS). Table S2. The methodological quality of cohort studies in accordance with the Newcastle-Ottawa Scale (NOS). Table S3. The methodological quality of case–control studies in accordance with the Newcastle-Ottawa Scale (NOS). (DOCX 19 kb) [file 12891_2017_1456_MOESM3_ESM.docx]

Supplementary table 1 The methodological quality of cross-sectional studies in accordance with the Newcastle-Ottawa Scale (NOS)

| Study | Design | Data collection | Response rate | Representativeness | Object and method | Power of testing | Statistical method | Total score |
| --- | --- | --- | --- | --- | --- | --- | --- | --- |
| Tang 1998 [44] | 1 | 1 | 0 | 1 | 1 | 0 | 1 | 5 |
| Kiyohara 1999 [32] | 1 | 1 | 1 | 0 | 1 | 0 | 1 | 5 |
| Choi 2007 [34] | 1 | 1 | 0 | 1 | 1 | 0 | 1 | 5 |
| Haldar  2007 [50] | 1 | 1 | 0 | 0 | 0 | 0 | 1 | 3 |
| Yu 2010 [51] | 1 | 1 | 1 | 1 | 1 | 0 | 1 | 6 |
| Chang 2012 [47] | 1 | 1 | 1 | 0 | 1 | 0 | 1 | 5 |
| Teng 2013 [36] | 1 | 1 | 1 | 1 | 1 | 0 | 1 | 6 |
| Bae 2015 [37] | 1 | 1 | 0 | 1 | 1 | 1 | 1 | 6 |
| Li  2015 [38] | 1 | 1 | 1 | 1 | 0 | 0 | 1 | 5 |
| Chatzistamatiou  2015 [49] | 1 | 1 | 0 | 0 | 1 | 0 | 1 | 4 |

Supplementary table 2 The methodological quality of cohort studies in accordance with the Newcastle-Ottawa Scale (NOS)

| Study | Selection | | | | Comparability | Outcome | | | Total score |
| --- | --- | --- | --- | --- | --- | --- | --- | --- | --- |
|  | Representativeness of the exposed cohort | Selection of the non-exposed cohort | Ascertainment of exposure | Demonstration that outcome of interest was not present at start of study | Comparability of cohorts on the basis of the design or analysis | Assessment of outcome | Was follow-up long enough for outcomes to occur? | Adequacy of follow up of cohorts? |  |
| David Curb  1986 [48] | 1 | 1 | 1 | 0 | 1 | 0 | 1 | 1 | 6 |
| Choi  2007 [33] | 0 | 1 | 1 | 1 | 2 | 1 | 1 | 1 | 8 |
| Choi  2010 [35] | 0 | 1 | 1 | 1 | 2 | 1 | 1 | 1 | 8 |
| Tian 2016 [46] | 0 | 1 | 1 | 1 | 1 | 1 | 1 | 1 | 7 |

Supplementary table 3 The methodological quality of case-control studies in accordance with the Newcastle-Ottawa Scale (NOS)

| Study | Selection | | | | Comparability | Exposure | | | Total score |
| --- | --- | --- | --- | --- | --- | --- | --- | --- | --- |
|  | Is the case definition adequate | Representativeness of the cases | Selection of Controls | Definition of Controls | Comparability of cases and controls on the basis of the design or analysis | Ascertainment of exposure | Same method of ascertainment for cases and controls | Non-Response rate |  |
| Yuan  2000 [45] | 1 | 1 | 0 | 0 | 1 | 0 | 1 | 1 | 5 |
